# Supplementary material for: Acinetobacter baumannii and Cefiderocol, between Cidality and Adaptability
Source: Microbiol Spectr. 2022 Sep 29;10(5):e02347-22. doi: 10.1128/spectrum.02347-22 (PMC9603721; doi:10.1128/spectrum.02347-22)
Supplement: Supplemental file 1 — Fig. S1 and S2 and Tables S1 and S2. Download spectrum.02347-22-s0001.pdf, PDF file, 0.3 MB [file spectrum.02347-22-s0001.pdf]

Figure S1. Population analysis profile of study sample

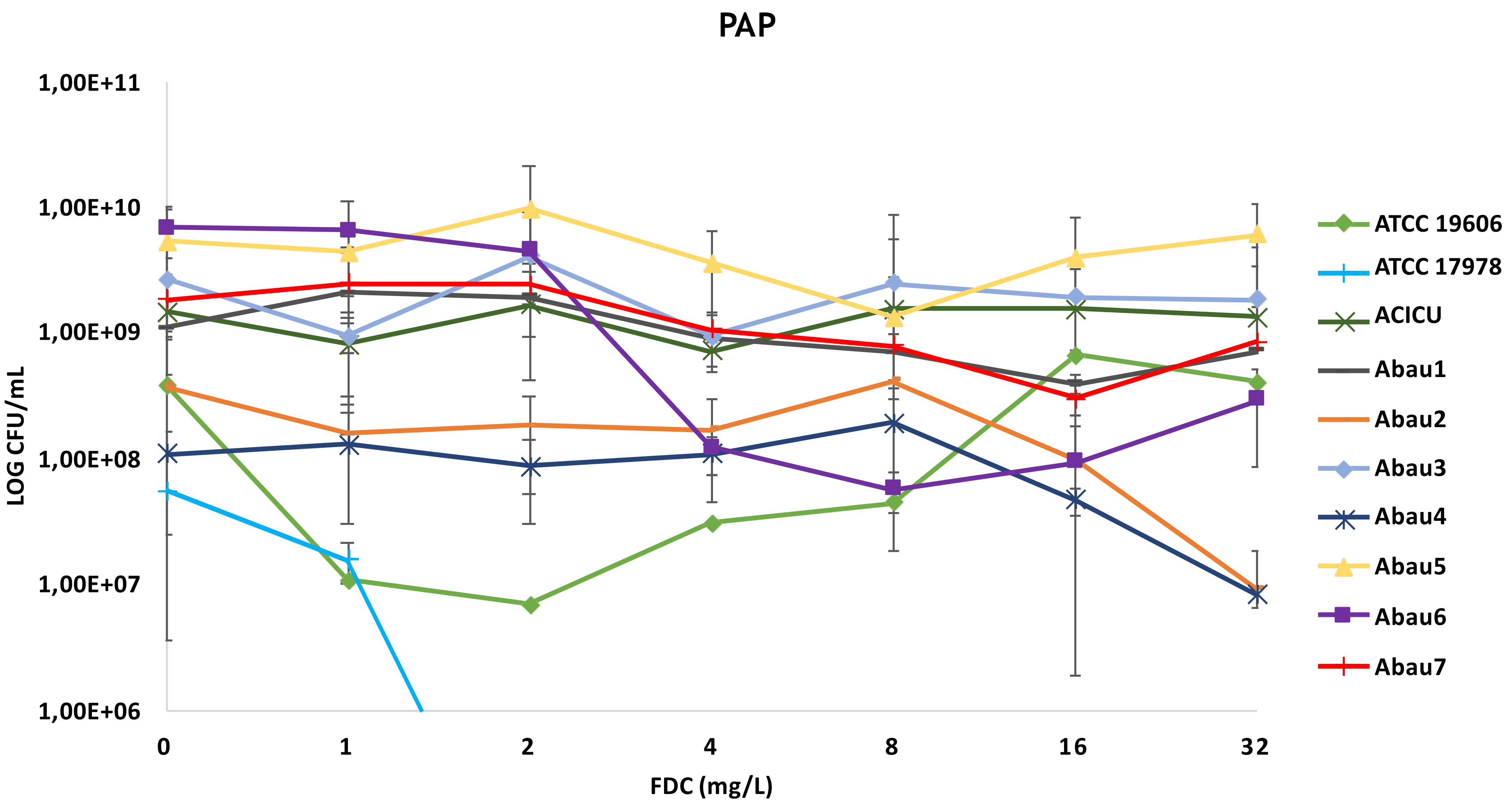

Figure S2. 2mg·L^{-1} cefiderocol/50% free CFU/mL ratio from PAP agar plates

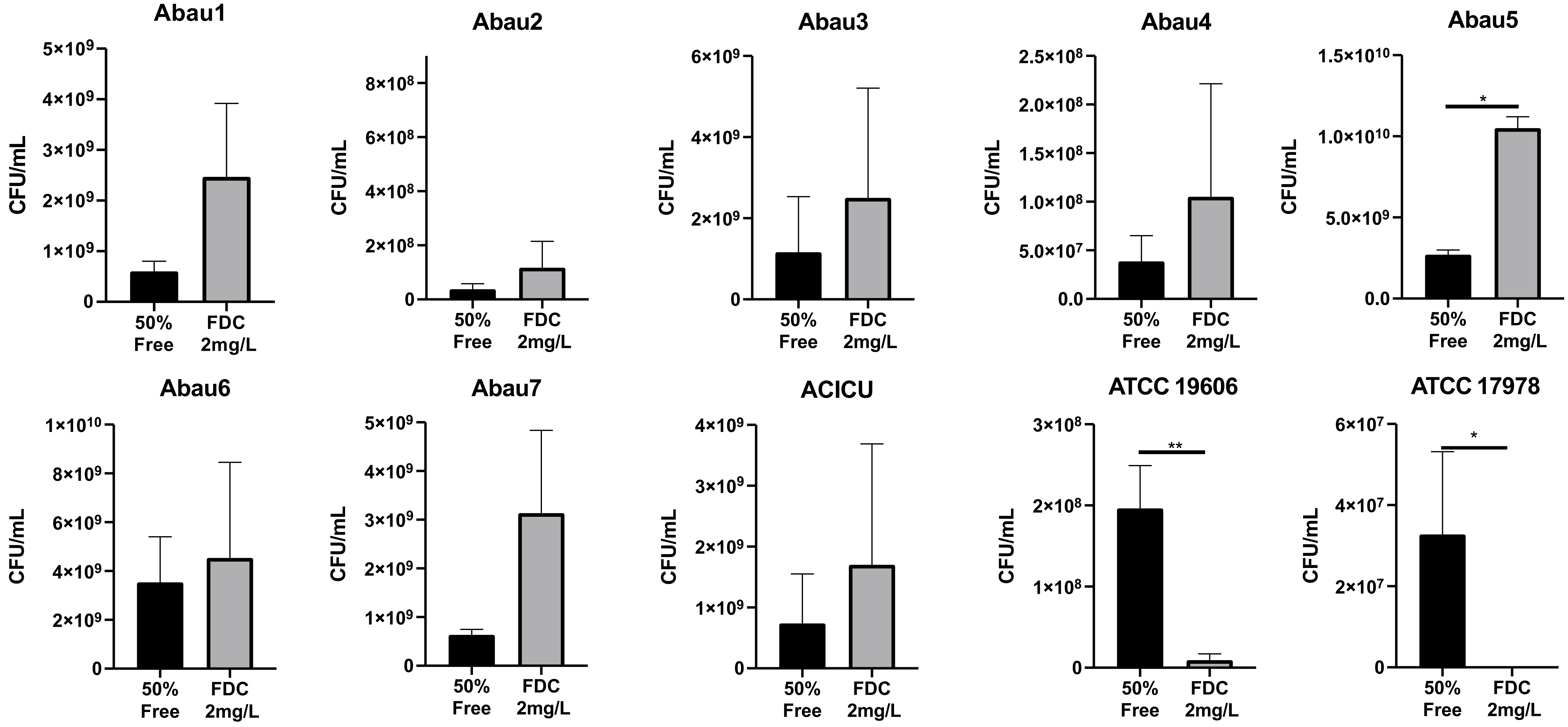

* *p*-value <0.05

** *p*-value <0.01

Table S1. *pbp*3/PBP3 and *ton*B/TonB SNPs and amino acid substitutions of the study sample

a) *pbp*3/PBP3

Strain	SNP	Amino acid substitution	Strain	SNP	Amino acid substitution
Abau1 Abau3 Abau4 Abau5 Abau6 Abau7	A387G	/	ACICU	A105	/
	G390A	/		C192T	/
	C441T	/		A387G	/
	C762T	/		G390A	/
	T768A	/		C441T	/
	C780T	/		T483C	/
	A906G	/		T645G	/
	C1134T	/		T768A	/
	C1218T	/		G867A	/
	C1410T	/		G870A	/
	A1566T	/		C1037T	A346V
	C1710T	/		C1108T	H370Y
Abau2	A387G	/		C1134T	/
	G390A	/		C1218T	/
	C441T	/		C1410T	/
	C762T	/		A1602G	/
	T768A	/		C1710T	/
	C780T	/	ATCC19606	C441T	/
	A906G	/		C762T	/
	C1134T	/		T981G	/
	C1218T	/		T1083C	/
	C1410T	/		C1134T	/
	C1544T	A515V		A1197G	/
	A1566T	/		C1218T	/
	C1710T	/		C1410T	/
				C1710T	/

b) *ton*B/TonB

Strain	SNP	Amino acid substitution
Abau1 Abau2 Abau3 Abau4 Abau5 Abau6 Abau7 ACICU	A173G	H58R
	A189G	/
	T213C	/
	C219T	/
	A237G	/
	C248A	A83E
	C480T	/
	A531G	/
	A561G	/
	G573A	/
ATCC 19606*	A555G	/
	A786G	/
	T803C	V268A
	A822G	/
	G825A	/

*ATCC 19606 *ton*B gene has all the SNPs reported for the other strains plus 5 peculiar SNPs

Table S2. Combined effect of FDC and BLIs

	FDC free MHA		FDC 1 mg/L MHA		FDC free MHA		FDC 1 mg/L MHA	
Strain	CAZ (mm)	CZA (mm)	CAZ (mm)	CZA (mm)	AMP (mg/L)	SAM (mg/L)	AMP (mg/L)	SAM (mg/L)
Abau1	6*	6	6	6	>256	>256	>256	>256
Abau3	6	6	6	22	>256	>256	>256	≤0.016
Abau5	6	6	6	6	>256	>256	>256	0.016
Abau6	6	6	6	6	>256	>256	>256	≤0.016
Abau7	6	6	6	18	>256	>256	>256	≤0.016
ACICU	6	6	6	6	>256	>256	>256	>256

MHA: Mueller-Hinton agar
FDC: cefiderocol
CAZ: ceftazidime
CZA: ceftazidime/avibactam
AMP: apmicillin
SAM: ampcillin/sulbactam
*as suggested by the disk diffusion manufacturers, a value of 6 mm was assigned every time there was no halo around the disk
